# Comparison of open surgery versus endoscopic-assisted release for gluteal muscle contracture: a systematic review and meta-analysis

**DOI:** 10.1186/s13018-023-04452-7

**Published:** 2024-01-06

**Authors:** YaMeng Jiang, Tao Li, Li Wang, Gang Chen, Jian Li

**Affiliations:** Department of Orthopedics, Orthopedic Research Institute, West China Hospital, Sichuan University, Chengdu, China

**Keywords:** Gluteal muscle contracture, Open release, Arthroscopic release

## Abstract

**Purpose:**

This study aimed to perform a systematic review and meta-analysis to compare the clinical outcomes of open surgery and arthroscopic release in gluteal muscle contracture (GMC).

**Methods:**

Two independent reviewers YM and WL conducted a systematic search of PubMed, Embase, Cochrane Library, and Web of Science to identify clinical trials that adhered to the PRISMA guidelines (Appendix A), spanning from inception to July 2023. Search items included ((“gluteal” OR “gluteus”) AND (“contracture” OR “fibrosis”)). Research comparing open surgery or arthroscopic release was included. Clinical outcomes were compared using the risk ratio for dichotomous variables and the standardized mean difference for continuous variables. A P value < 0.05 was deemed statistically significant.

**Results:**

Four studies with 453 patients met the selection criteria and were included in this review. Compared with open surgery, in the case of similar postoperative functional satisfaction (1.21, 95% CI = 0.46–3.17, P = 0.70), the arthroscopic release achieved advantages in postoperative complications (3.5, 95% CI = 1.75–7.03, P = 0.0004), cosmetic satisfaction (0.07, 95% CI = 0.01–0.65, P = 0.02), length size (5.65, 95% CI = 4.11–7.19, P < 0.001), and hospitalization duration (1.57, 95% CI = 0.89 to 2.26, P < 0.001).

**Conclusion:**

This research shows that both open surgery and arthroscopic release improve functional satisfaction. The arthroscopic release could result in fewer complications, better cosmetic satisfaction, shorter length size, and shorter hospitalization duration.

*Registration and protocol*

There is no registration and protocol for this meta-analysis.

## Introduction

Gluteal muscle contracture (GMC) is a clinical syndrome characterized by contracture of gluteal muscles, tensor fascia lata (TFL), iliotibial band (ITB), and related fascia [1–5]. In severe cases, GMC can involve the hip external; it exists globally but is more prevalent in China, with an overall childhood incidence rate of 1–2.5% [6, 7]. The etiology of GMC may be related to intramuscular antibiotics and antimalarial drugs such as quinine [8]. In addition to intramuscular injections, causes of such contractures include trauma, injury of the brachial plexus during birth, and congenital abnormalities [9].

Patients diagnosed with GMC are commonly associated with abduction, external rotation, and limited flexion and adduction of the affected hip [1, 10]. The other features include a positive cross sign, squatting test, and Ober’s sign [6]. The pathognomonic presentation of GMC can lead to substantial functional limitations for patients’ daily activities, which will persist throughout their life without proper treatment.

For a long time, the release has been considered the standard treatment method for patients with GMC [6, 11]. In the conventional open out of the fibrotic bands, the patient is placed in the lateral decubitus position, with varied incision placements ranging from just posterior to the greater trochanter to directly over the buttock. [12] It is introduced in all established cases but is highly recommended for severe cases if a wide incision provides precise exposure and complete release, protecting the sciatic nerve [6]. However, the high rates of complications such as extensive tissue manipulation, hematoma formation, wound complications, and slow recovery time have been critical in decreased patient satisfaction [13, 14]. These factors have been crucial in reducing fallen patient satisfaction. The widespread adoption of arthroscopic release allows for minor surgical trauma, earlier rehabilitation, shorter operative time, and fewer postsurgical complications [6]. The arthroscopic release also successfully meets the esthetic requirements of patients. However, there are few previous studies comparing the surgical outcomes of these two operations [12, 15]. This study conducts a systematic review and meta-analysis to compare the clinical outcomes and rate of complications of open surgery and arthroscopic release.

## Methods

### Search strategy

Two independent reviewers YM and WL performed this systematic review and meta-analysis of the PubMed, Embase, Web of Science, and Cochrane databases from July 2023. The search strategy used was ((“gluteal” OR “gluteus”) AND (“contracture” OR “fibrosis”)). The keywords were restricted to the title or abstract. The senior author LT, a hip surgeon, reviewed discrepancies in the inclusion or exclusion of studies.

### Selection criteria

The inclusion criteria were as follows: (1) clinical trials of gluteal muscle contracture comparing the clinical effects of formal conventional open and arthroscopic release; (2) published in a peer-reviewed journal; (3) published in English. The exclusion criteria included the following: (1) animal studies, abstracts, reviews, or conference proceedings; (2) incomplete data. Based on the inclusion and exclusion criteria, the titles and abstracts of each article were screened by LT, and the full texts were subsequently reviewed by YM, LT and WL.

### Data extraction

Studies with potentially relevant titles were selected for further review of the abstracts. Papers that satisfied the inclusion criteria were retrieved for full-text assessment. After a more detailed analysis of documents addressing surgical outcomes, the following data were extracted from these articles by YM: population and mean ages of the patients, the number of male and female patients in each study, types of treatment, surgical duration, length of incision, postsurgical off-bed activities time, hospitalization duration, follow-up time and numbers of patients, complications, as well as functional and cosmetic satisfaction.

### Assessment of methodological quality

The Newcastle–Ottawa Scale (NOS) was performed by YM to assess the quality of nonrandomized studies in the meta-analysis. The scoring system summarized eight aspects of each study: case definition adequacy, representativeness of the cases, selection of controls, the definition of controls, comparability of cases and controls based on the design or analysis, ascertainment of exposure, the same method of ascertainment for cases and controls, and comparison of nonresponse rate between cases and controls. High-quality studies were defined as a score of six or more of 9 total points.

### Data synthesis and analysis

The primary outcomes encompass cosmetic satisfaction, functional satisfaction, as well as complications and recurrence. Hospitalization time and length of incision are considered as the second outcomes. Review Manager 5.3 was used by YM and WL to perform statistical analysis. Discontinuous data such as cosmetic and functional satisfaction were tested by the M–H and variance homogeneity tests, with a 95% study confidence interval and 95% total confidence interval. Continuous outcomes were calculated and expressed as the standardized mean difference (SMD), and dichotomous outcomes were expressed as the risk ratio (RR). Heterogeneity between studies was quantified using the I^2^ statistic. I^2^ values of 25%, 50%, and 75% indicated low, medium, and high heterogeneity, respectively. The fixed-effect model was used when I^2^ < 50%; otherwise, the random-effect model was used. *High heterogeneity was analyzed through the exclusion of one or two studies for each outcome.*

## Results

### Literature search

**Fig. 1:**
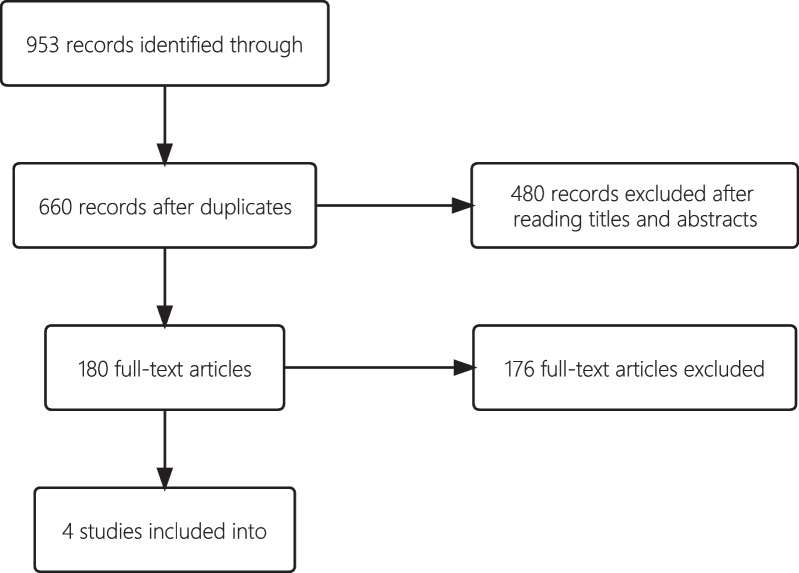
Literature review search process

A total of 953 articles were selected after the initial search (PubMed = 291, Embase = 457, WOS = 185, Cochrane = 20), and 660 articles remained after duplicates were removed. A total of 480 were excluded after a review of the titles and abstracts. Then, the abstracts of the remaining 180 papers were carefully read, and 176 studies were excluded for not meeting the criteria (Fig. 1). Finally, 4 studies retrospectively assessed the surgical outcomes of traditional open and arthroscopic releases were included.

### Study characteristics

Four studies with 453 patients were included. All included studies were Level III retrospective studies with a mean follow-up time of 24.353 months. All the included studies compared open surgery and arthroscopic release plus GMC. Table 1 shows the characteristics and patient demographics of the 4 included studies. The assessment of the study quality is shown in Table 2.

**Table 1:** Characteristics of included studies

Author	Year	Region	Funding	Level of evidence	No of patients		Mean age (yr)		SEX		Mean follow-up (mo)
					Open	Art.	Open	Art.	Male	Female	
Fu et al.	2011	China	NSFC	3	50	52	8.9(6~19)	9.2(5-20)	44	58	26(12~24)
Dai et al.	2017	China	NSFC and NSFH	3	44	48	20.6(17~35)	20.8(18~28)	34	86	24
Rai et al.	2017	China	NSFC	3	71	75	25.30(17~42)	25.05(16~46)	58	88	22
Zhang et al.	2018	China	No	3	72	41	22.39±3.80	23.05±4.67	57	56	26.76(open), 25.2(Art.)

**Table 2:** Quality accessing of included articles by using the Newcastle–Ottawa Scale

Study	Year	Selection				Comparability		Outcome			Quality score
		Representativeness of the exposed cohort	Selection of the non-exposed cohort	Ascertainment of exposure	Demonstration that outcome of interest was not present at start of study	Comparability of cohorts on the basis of the design or analysis	Comparability of cohorts on the basis of the measurement	Assessment of outcome	Was follow-up long enough for outcomes to occur	Adequacy of follow-up of cohorts	
Fu et al	2011	-	☆	★	-	★	★	-	★	★	5.5
Dai et al	2017	-	☆	★	-	★	★	★	★	★	6.5
Rai et al	2017	-	☆	★	-	★	★	-	★	-	4.5
Zhang et al	2018	-	☆	★	-	★	★	-	★	★	5.5

### Quality assessment

One study received a rating of 4.5, two were rated as 5.5 and one study earned a score of 6.5, indicating the overall high quality of the studies included.

### Meta-analysis

Functional satisfaction, rate of complications and recurrence, cosmetic satisfaction, length of incision, and hospitalization duration are included in the meta-analysis.

#### Functional satisfaction

**Fig. 2:**
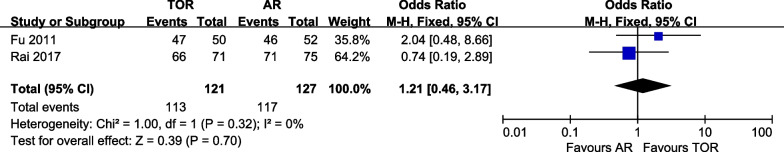
Forest plot of functional satisfaction

All the included studies reported that the two methods resulted in similar functional outcomes. Data from two of the studies can be used for meta-analysis. In the overall analysis of these two studies, the reasonable satisfaction between conventional open release and arthroscopic release shows no significant difference (1.21, 95% CI is 0.46 to 3.17, the P value is 0.70), with a low heterogeneity (I^2^ = 0%, the P value is 0.32) (Fig. 2).

#### Complications and recurrence

**Fig. 3:**
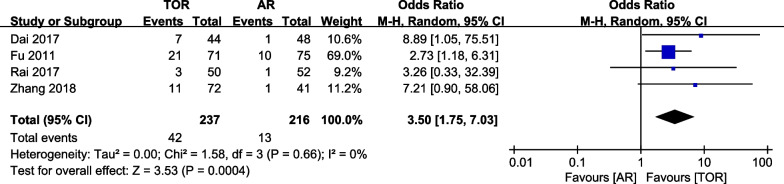
Forest plot of complications

**Fig. 4:**
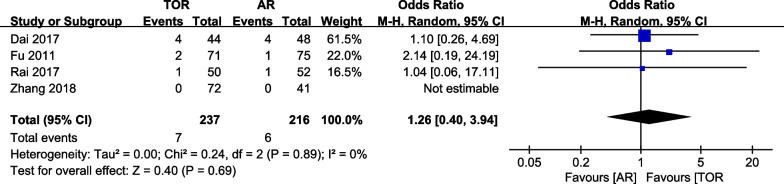
Forest plot of recurrence

All the included studies reported complications and recurrence (Table 3). In the meta-analysis. The rate of complications of arthroscopic release is significantly lower than that of open surgery (3.50, 95% CI is 1.75 to 7.03, the P value is 0.0004). The heterogeneity is low (I^2 ^= 0%, the P values is 0.66) (Fig. 3). There is no significant difference in the comparison of recurrence (1.26, 95% CI is 0.40 to 3.94, the P value is 0.69) (Fig. 4). The heterogeneity is low (I^2 ^= 0%, the P value is 0.89).

**Table 3:** Complications and Recurrence rates of included studies

Author	Complications		Recurrence	
	Conventional open release	Arthroscopic release	Conventional open release	Arthroscopic release
Fu et al.	3 (50)	1 (52)	1 (50)	1 (52)
Dai et al.	7 (44)	1 (48)	4 (44)	4 (48)
Rai et al.	21 (71)	10 (75)	2 (71)	1 (75)
Zhang et al.	10 (72)	1 (41)	1 (72)	0 (41)
Total	42 (237, 17.72%)	13 (216, 6.02%)	8 (237, 3.38%)	6 (216, 2.78%)

#### Cosmetic satisfaction

**Fig. 5:**
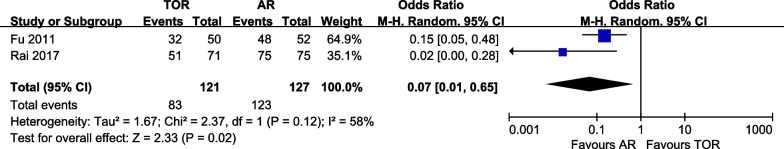
Forest plot of cosmetic satisfaction

Two studies measured cosmetic satisfaction. In the overall analysis of these two studies, arthroscopic release led to a higher percentage of identifying cosmetic satisfaction than conventional open release (0.07, 95% CI is 0.01 to 0.65, the P value is 0.02), with high heterogeneity between studies (I^2^ = 58%, the P value is 0.12) (Fig. 5).

#### Length of incision

**Fig. 6:**
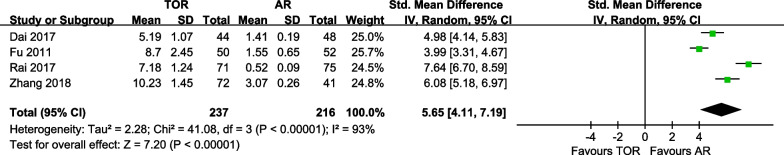
Forest plot of incisions size

**Fig. 7:**
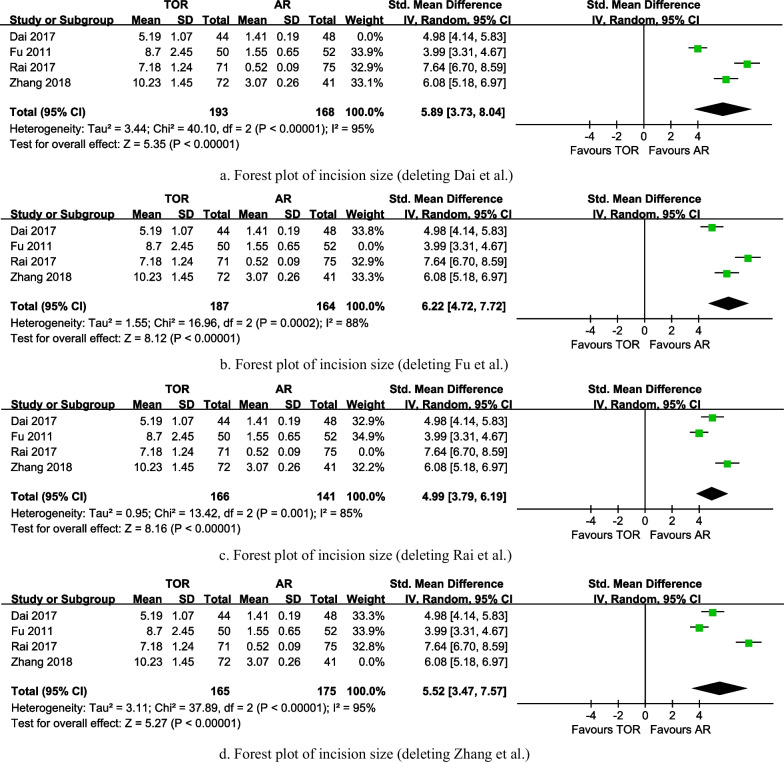
**a** Forest plot of incision size (deleting Dai et al.). **b** Forest plot of incision size (deleting Fu et al.). **c** Forest plot of incision size (deleting Rai et al.). **d** Forest plot of incision size (deleting Zhang et al.)

Four studies analyzed the incision length of the patients and provided detailed statistics. The overall analysis of incision size shows that arthroscopic release has a smaller incision length than conventional open release (5.65, 95% CI is 4.11 to 7.19, the P value is less than 0.001), with high heterogeneity (I^2^ = 93%, the P value is less than 0.001) (Fig. 6). After excluding either the Fu et al. or Rai et al. study, heterogeneity decreased but remained high (I^2^ = 88%, the P value is less than 0.001; I^2^ = 85%, the P value is 0.001). Conversely, the removal of the Dai et al. or Zhang et al. study resulted in increased heterogeneity (I^2^ = 95%, the P value is less than 0.001; I^2^ = 95%, the P value is less than 0.001). In all cases, the outcomes continued to favor the arthroscopic release group (6.22, 95% CI is 4.72 to 7.72, the P value is less than 0.001; 4.99, 95% CI is 3.79 to 6.19, the P value is less than 0.001; 5.89, 95% CI is 3.73 to 8.04, the P value is less than 0.001; 5.52, 95% CI is 3.47 to 7.57, the P value is less than 0.001) (Fig. 7).

#### Hospitalization duration

**Fig. 8:**
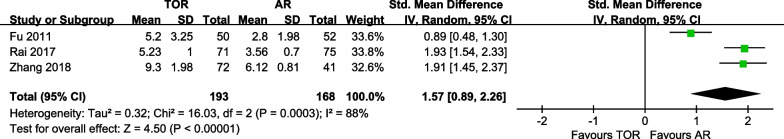
Forest plot of hospitalization duration

**Fig. 9:**
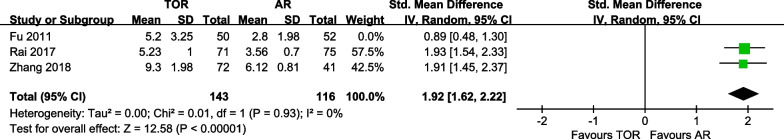
Forest plot of hospitalization duration (deleting Fu et al.)

For hospitalization duration, three articles provided clear statistics. In the overall analysis of the hospitalization duration of these three articles, the arthroscopic group took less hospitalization time than the conventional open group (1.57, 95% CI is 0.89 to 2.26, the P value is less than 0.001), with high heterogeneity (I^2^ = 88%, the P value is less than 0.001) (Fig. 8). After deleting Fu et al. study, the heterogeneity decreases (I^2^ = 0%, the P value is 0.93) (Fig. 9).

## Discussion

The most important finding of this study was that the arthroscopic release group had a lower probability of clinical complications and other better clinical outcomes when the two methods achieved similar functional outcomes. Previous studies comparing surgical outcomes between conventional open waivers and arthroscopic release have largely shown that arthroscopic release yields comparable results to traditional release. Nonetheless, it is important to highlight that arthroscopic release may have constraints in managing severe cases, as it may not effectively prevent sciatic nerve damage or achieve the comprehensive therapeutic outcome provided by open surgery [6, 10–12, 14, 16]. People haven’t agreed on comparing complications and recurrence rates between the two operations. Although one article performed a systematic review to compare conventional open release and arthroscopic release, it was a qualitative rather than a quantitative analysis [12].

In this meta-analysis, the arthroscopic release of the included four articles showed no significant difference with conventional open release in functional satisfaction and even performed better in cosmetic satisfaction. Regarding the length of incisions and hospitalization duration, arthroscopic release is better than conventional open release. The subjective surgery outcomes also suggested the equality of these two operations. All four articles measured surgical outcomes through a variety of methods [10, 11, 14, 17]. Both Fu et al. and Zhang et al. used excellent, good, acceptable, and poor to describe the subjective effect of surgical treatment [11, 14]. In Fu et al.’s article, which used Wang et al.’s criteria [18], there were 32 excellent, 15 good, two acceptable, and one poor in the conventional open group; in contrast, there were 34 perfect, 12 good, one fair, and five poor in the endoscopic group [11]. In Zhang et al.’s study, which used Liu et al.’s criteria, there were 60 excellent, six good, five fair, and one poor in the conventional open group; in contrast, in the endoscopic group, there were 33 perfect, five good, three acceptable, and no poor [14]. Generally, there is no significant difference between the conventional and endoscopic release groups. However, in Fu et al.’s study, the patients in the arthroscopic group seemed to be more likely to score poorly than those in the conventional open group. We think the operation time of this trial may contribute to this result. It took place between 2006 and 2008, so the operator in this trial may not have been as good at endoscopic release therapy, resulting in higher rates of poor scores.

The results above show that, in the case of similar functional outcomes, compared with conventional open release therapy, arthroscopic release achieved higher advantages in cosmetic satisfaction, length size, hospitalization duration, postoperative complications and lower rate of complications. These conclusions may support arthroscopic release when deciding gluteal muscle contracture therapy, leading to a better postsurgical outcome with a higher possibility. Arthroscopic release is a minimally invasive technique and due to its smaller incisions and reduced tissue damage, the resulting scars are usually smaller and less noticeable. These benefits often lead to faster recovery times and shorter hospital stays. The minimized tissue trauma might also lower the risk of certain complications, such as infections or scar formation. Combined with the scoping review of Kay et al [19]. about arthroscopic management of greater trochanter pain syndrome (GTPS), which includes GMC, advocate for the ongoing application and exploration of arthroscopic approaches to GTPS, laying the groundwork for more extensive prospective studies in future to validate relevant findings. From a quantitative analysis perspective, this study further validates the applicability of this viewpoint in GMC.

This study measured objective surgical outcomes by meta-analysis compared to previous studies, which only performed a systematic review and focused more on other aspects of gluteal muscle contracture. Additionally, our study measures surgical outcomes from multiple perspectives, ranging from cosmetic and functional satisfaction to postsurgical complications. Meanwhile, there are several limitations to our study. First, the number of included studies is inadequate, resulting in small numbers of included studies for each outcome, but the total sample size is 453. Furthermore, the heterogeneity of some outcomes is high. It cannot be eliminated through sensitivity analysis or subgroup analysis. In addition, all studies were conducted in China, reflecting regional bias.

## Conclusion

This study supports that arthroscopic GMC release causes less trauma, is less invasive, has fewer surgical complications, and achieves the same release effect as open surgery.

## Data Availability

The data extracted from the included studies and utilized for all analyses in this review can be found within the four referenced published articles (https://journals.sagepub.com. https://doi.org/10.1177/1120700017754013, https://journals.lww.com/pedorthopaedics/abstract/2011/07000/comparison_of_endoscopic_surgery_and_open_surgery.24.aspx, https://doi.org/10.1186/s12891-017-1484-6, https://www.ncbi.nlm.nih.gov/pmc/articles/PMC5995040/). Data analysis and analytic code were performed using Review Manager 5.3.
